# Intrapericardial cardiosphere-derived cells hinder epicardial dense scar expansion and promote electrical homogeneity in a porcine post-infarction model

**DOI:** 10.3389/fphys.2022.1041348

**Published:** 2022-11-15

**Authors:** Alejandro Carta-Bergaz, Gonzalo R. Ríos-Muñoz, Verónica Crisóstomo, Francisco M. Sánchez-Margallo, María J. Ledesma-Carbayo, Javier Bermejo-Thomas, Francisco Fernández-Avilés, Ángel Arenal-Maíz

**Affiliations:** ^1^ Gregorio Marañón Health Research Institute (IiSGM), Department of Cardiology, Hospital General Universitario Gregorio Marañón, Madrid, Spain; ^2^ Centre for Biomedical Research in Cardiovascular Disease Network (CIBERCV), Madrid, Spain; ^3^ Department of Bioengineering and Space Engineering, Universidad Carlos III de Madrid, Madrid, Spain; ^4^ Jesús Usón Minimally Invasive Surgery Centre, Cáceres, Spain; ^5^ Biomedical Image Technologies, ETSI Telecomunicación, Universidad Politécnica de Madrid, Madrid, Spain; ^6^ CIBER-BBN, Instituto de Salud Carlos III, Madrid, Spain; ^7^ Medical School, Universidad Complutense de Madrid, Madrid, Spain

**Keywords:** ventricular tachycardia, epicardial arrhythmic substrate, ischemic scar, cardiosphere-derived cell, cellular therapy

## Abstract

The arrhythmic substrate of ventricular tachycardias in many structural heart diseases is located in the epicardium, often resulting in poor outcomes with currently available therapies. Cardiosphere-derived cells (CDCs) have been shown to modify myocardial scarring. A total of 19 Large White pigs were infarcted by occlusion of the mid-left anterior descending coronary artery for 150 min. Baseline cardiac magnetic resonance (CMR) imaging with late gadolinium enhancement sequences was obtained 4 weeks post-infarction and pigs were randomized to a treatment group (intrapericardial administration of 300,000 allogeneic CDCs/kg), (*n* = 10) and to a control group (*n* = 9). A second CMR and high-density endocardial electroanatomical mapping were performed at 16 weeks post-infarction. After the electrophysiological study, pigs were sacrificed and epicardial optical mapping and histological studies of the heterogeneous tissue of the endocardial and epicardial scars were performed. In comparison with control conditions, intrapericardial CDCs reduced the growth of epicardial dense scar and epicardial electrical heterogeneity. The relative differences in conduction velocity and action potential duration between healthy myocardium and heterogeneous tissue were significantly smaller in the CDC-treated group than in the control group. The lower electrical heterogeneity coincides with heterogeneous tissue with less fibrosis, better cardiomyocyte viability, and a greater quantity and better polarity of connexin 43. At the endocardial level, no differences were detected between groups. Intrapericardial CDCs produce anatomical and functional changes in the epicardial arrhythmic substrate, which could have an anti-arrhythmic effect.

## Introduction

Ventricular arrhythmias are responsible for more than half of sudden deaths in developed countries. Survivors of an aborted cardiac death from sustained monomorphic ventricular tachycardia (VT) are at an increased risk of a new sudden cardiac death due to VT recurrence (Zeppenfeld et al., 2022). In this scenario, implantable cardiac defibrillators (ICDs) have shown to reduce the mortality (Kuck et al., 2000; Connolly et al., 2000a; Connolly et al., 2000b; Antiarrhythmics versus Implantable Defibrillators (AVID) Investigators, 1997; Dinov et al., 2014). However, ICDs are not a cure for VTs because they do not modify the arrhythmic substrate. In fact, appropriate ICD shocks are associated with an increased mortality. Catheter ablation has shown to reduce the incidence of ICD therapies, although recurrence of the VT is not uncommon (Dinov et al., 2014; Zeppenfeld et al., 2022). Although the formation of new circuits may explain some recurrences, the presence of deep substrate far from the reach of the radiofrequency catheter seems to justify most of the VT recurrences (Liuba et al., 2014; Proietti et al., 2015; Siontis et al., 2016).

Patients with ischemic and nonischemic cardiomyopathy exhibit myocardial scars that can expand to the epicardium and sustain VT circuits. Since mapping and ablation of the epicardial substrate are often challenging, therapies to prevent the epicardial expansion of scars are needed (Tung et al., 2013; Aryana et al., 2020). Clinical studies have shown that cardiac stem cells can modify the ischemic scar and prevent postinfarction remodeling through a paracrine effect (Chugh et al., 2012; Malliaras et al., 2014; Makkar et al., 2020).

This study hypothesizes that intrapericardial administration of allogenic cardiosphere-derived cells (CDCs) can modify the electrical and structural remodeling of the epicardial scar. Compared to other routes of administration (intracoronary, surgical, intramyocardial), intrapericardial administration does not affect the coronary flow and produces more homogeneous and durable cell retention (Blázquez et al., 2016).

## Methods

### Study design

**FIGURE 1:**
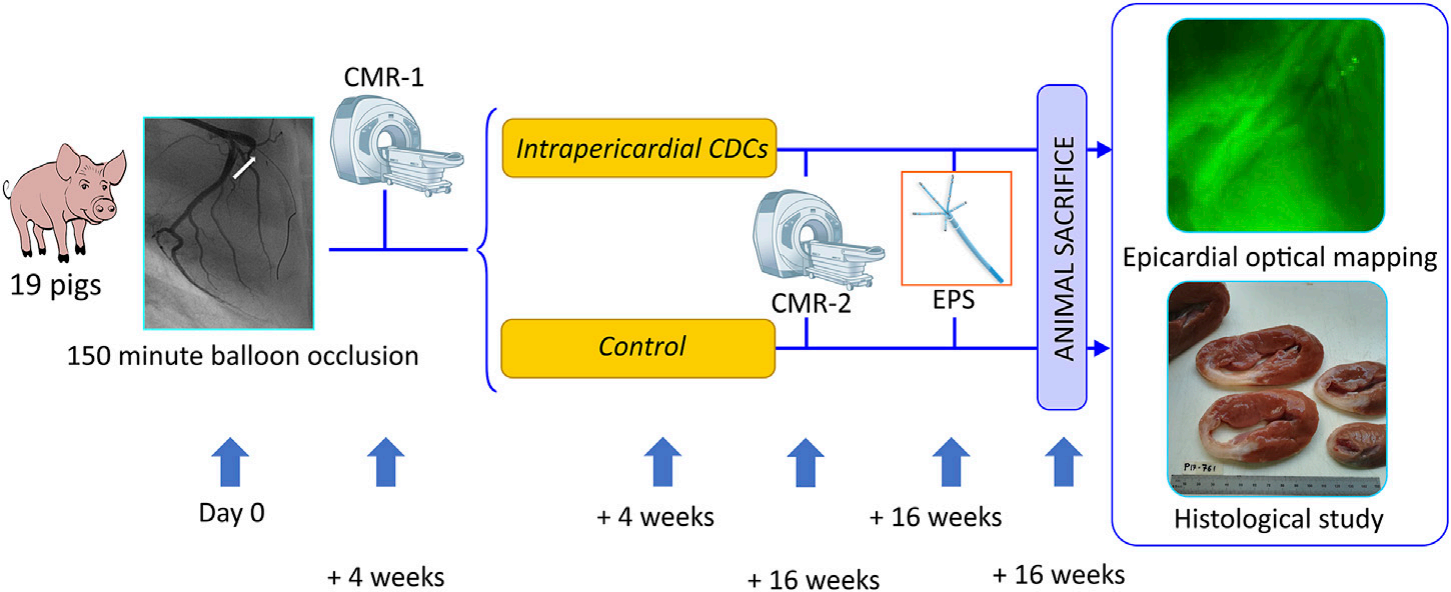
Study protocol. Nineteen Large White pigs with an induced anterior myocardial infarction were randomized to receive intrapericardial CDCs or to a sham procedure. Abbrv: CDC = cardiosphere-derived cells, CMR = cardiac magnetic resonance, EPS = electrophysiologic study.

A double-blind, randomized, placebo-controlled study was conducted using a porcine post-infarction experimental model to assess the effect of intrapericardial allogeneic CDCs on the epicardial arrhythmic substrate. We studied 19 pigs following the protocol described in Figure 1. The study was carried out at the Jesús Usón Minimally Invasive Surgery Centre (Cáceres, Spain), certified to conduct experimental studies with laboratory animals in accordance with European legislation (European Directive 2010/63/EU). The study conforms to the Guide for the Care and Use of Laboratory Animals and was approved by the Ethics Committee of the researchers’ institution.

The animal species was chosen based on the similarity of the pig and human heart in terms of cardiovascular anatomy, ventricular function, cardiac metabolism, electrophysiology, distribution of the coronary artery system, and collateralization after an acute event (Dixon and Spinale, 2009; Kim et al., 2011; Lelovas et al., 2014). In addition, animals that survive the acute phase of the infarction develop transmural infarcts of similar size and location (Klein et al., 1984).

### Study protocol

All procedures were performed under general inhalation anesthesia using sevoflurane and pigs were mechanically ventilated using an orotracheal tube.

#### Induction of reperfused acute myocardial infarction

The experimental model of myocardial infarction-reperfusion that is described has already been used and has been extensively validated (Yoshimizu et al., 1986; Näslund et al., 1992; Kraitchman et al., 2000; Reffelmann et al., 2004; Krombach et al., 2005; Pérez de Prado et al., 2009). Compared to the occlusive infarction model, this infarction with reperfusion model offers the advantage of producing a more heterogeneous epicardial scar (Garcia-Dorado et al., 1987). Balloon occlusion of the mid left anterior descending artery, just distal to the first diagonal branch, was performed through percutaneous femoral access to achieve a Thrombolysis In Myocardial Infarction grade 0 flow for 150 min. Before occluding the artery, an IV bolus of lidocaine (1 mg/kg) was administered to prevent ischemia-induced ventricular arrhythmias. In the event animals presented ventricular tachyarrhythmias during the occlusion of the coronary artery, advanced cardiopulmonary resuscitation maneuvers were started following the resuscitation guidelines. After the infarction, pigs were started on aspirin 500 mg QD and clopidogrel 300 mg QD until their sacrifice and amiodarone 400 mg QD for 5 days postinfarction.

#### Cardiac magnetic resonance (CMR) studies

Two CMR studies were performed at 4 and 16 weeks after the induction of myocardial infarction with a 1.5 Tesla scanner (Intera, Philips, the Netherlands) using a 5-element dedicated cardiac coil. Following the same protocol that our working group has already used in previous works (Perez-David et al., 2011), the CMR study consisted of cine steady-state free-precession (SENSEx2, repetition time 2.4 ms, echo time 1.2 ms, average in-plane spatial resolution: 1.62 mm × 2 mm, 30 phases per cycle, 8-mm slice thickness without gap) and late gadolinium enhancement imaging (3D inversion-recovery turbo gradient echo sequence, pre-pulse delay optimized for maximal myocardial signal suppression; SENSE x2, flip angle 15°, repetition time 3.4 ms, echo time 1.3 ms, actual spatial resolution: 1.48 mm × 1.66 mm, interpolated spatial resolution 1.29 mm × 1.29 mm, 5-mm actual slice thickness, inversion time 200–300 ms; acquisition window was set to 150–170 ms). Images were obtained in short-axis views and 4-, 2-, and 3-chamber standard views. Late gadolinium enhancement images were obtained 10 min after injection of gadodiamide 0.2 mmol/kg. Cine and late gadolinium enhancement images were obtained to determine left ventricular end-diastolic volume (LVEDV), left ventricular end-systolic volume (LVESV), left ventricular ejection fraction (LVEF), total scar mass (TSM), and total myocardial mass (TMM) using specialized software (QMass MR version 7.0, MEDIS, the Netherlands). The scar was defined as those areas with voxel intensity values ≥2 standard deviations with respect to that of remote healthy myocardium (HM). (Yan et al., 2006; Perez-David et al., 2011).

Endocardial and epicardial scar growth was assessed by comparing the endocardial and epicardial signal intensity maps between the two CMR studies. Following the method previously published by our working group, signal intensity maps were obtained through the postprocessing of late gadolinium enhancement sequences, where the average signal intensity of the internal (endocardium) and external (epicardium) half of the myocardial thickness was projected into two separate 3D reconstructions (Perez-David et al., 2011; Arenal et al., 2014; Ávila et al., 2015). The left ventricular endo- and epicardial contours were defined on contiguous short-axis slices using the software QMass MR. Using custom software developed in the MATLAB environment (MathWorks, Natick, Massachusetts) 3D endo- and epicardial reconstructions were computed from an image volume integrating the short-axis, 4- and 2-chamber. The 3D visualization interface was implemented in Java (Sun Microsystems) using VTK (Kitware, Clifton Park, New York) visualization algorithms. Voxels with intensity values ≥2 but <3 were coded as heterogeneous tissue (HT), whereas voxels with values ≥3 were coded as dense scar (DS) (Yan et al., 2006; Perez-David et al., 2011). Dense scar and heterogeneous tissue were differentiated based on signal intensity, thus allowing the measurement of endocardial dense scar (EnDS), epicardial dense scar (EpDS), endocardial heterogeneous tissue (EnHT), and epicardial heterogeneous tissue (EpHT). The effect of CDCs was assessed by comparing the percentage change in EnDS, EpDS, EnHT, and EpHT. Each percentage change was calculated by dividing the difference of the variable between the first and second CMR by the value of the variable in the first CMR.

#### Isolation of allogeneic CDCs and intrapericardial administration

Four weeks after the creation of the myocardial infarction, animals were randomized to a treatment group (*n* = 10) that received intrapericardial allogenic CDCs (300,000 CDCs/kg in HypoThermosol solution) *via* an angiographically-guided subxiphoid puncture or to a control group (*n* = 9) where only the CDCs diluting solution was administered. CDCs were obtained from the hearts of other sacrificed healthy Large White pigs. Phenotypic and molecular characterization of CDCs were performed by flow cytometry and polymerase chain reaction, as detailed in previous works (Blázquez et al., 2016).

#### Endocardial electroanatomic mapping of the left ventricle

High-density electroanatomic mapping of the ventricle was performed with the CARTO3 system (Biosense Webster, CA, United States) using a PentaRay catheter (Biosense Webster, CA, United States) through retroaortic access. A filling density threshold of ≤5 mm was established for the regions with bipolar electrograms of amplitude ≤1.5 mV. Using the measurement tool included in CARTO3, the areas of total scar (bipolar electrograms ≤1.5 mV) and DS (≤0.5 mV) were calculated. Electrograms with amplitudes comprised between 0.5 and 1.5 mV were color-coded as HT.

#### Sacrifice

Following the guidelines of the American Veterinary Medical Association, once the electrophysiological study was completed, pigs were euthanized. Animals under deep general anesthesia were placed in a supine position on the surgical table. A sternotomy was performed to expose the heart and the great vessels. Following heparinization (unfractionated heparin, 1 mg/kg) and cardiac arrest after a rapid IV injection of potassium chloride, heart extraction was performed expeditiously. After explanting the heart, a cannula was inserted into the ascending aorta to retrogradely perfuse the heart with a cardioplegic solution at 4°C (pH 7.4; solution composition in mM: 140 NaCl; 5.4 KCl; 1 MgCl^2^; 5 HEPES buffer; 11 glucose; 1.8 CaCl_2_).

#### Epicardial optical mapping

**FIGURE 2:**
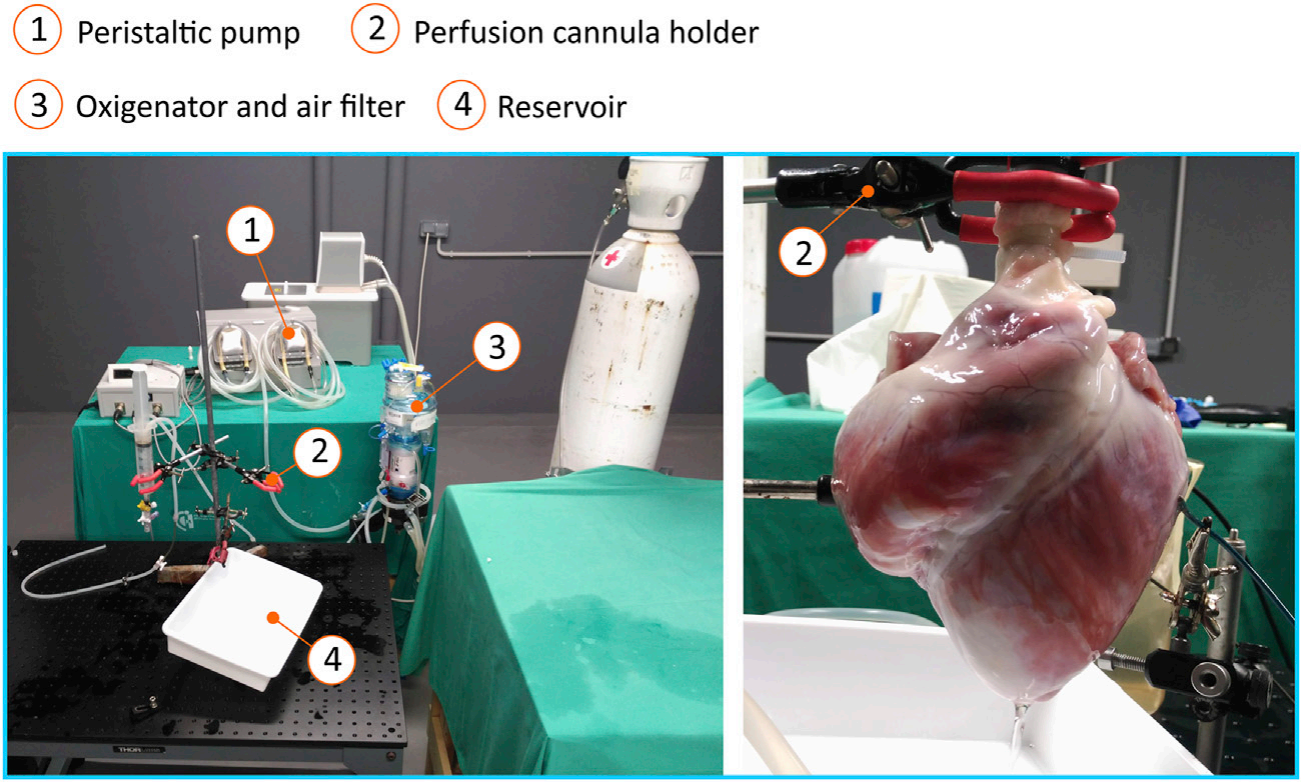
Langendorff perfusion system. The layout of the equipment (left) and the system used to suspend the heart (right) are shown.

For the epicardial optical mapping, the heart was removed from the cold solution and suspended from the aortic cannula to initiate retrograde warm perfusion with 1 L of modified Krebs solution at 36.5°C (pH 7.4; solution composition in mM: 120 NaCl; 25 NaHCO_3_; 1.8 CaCl_2_; 5.4 KCl; 1 MgCl*2*; 5.5 glucose; 1.2 H2PO_2_H_2_O). When the Krebs solution was clear of blood, the circuit was closed to enable recirculation. The Krebs solution was oxygenated by constant bubbling of carbogen gas. If the heart presented ventricular tachyarrhythmias when it was transferred to the Langendorf system (Figure 2), repeated 5 J shocks were administered with external paddles to return to normal sinus rhythm. A dose of 10 mm 2,3-butanedione monoxime (Biotium, Inc. Hayward, California, United States) was administered to achieve electromechanical dissociation and avoid hypoxia and motion artifacts during optical mapping. Despite there are other available excitation-contraction uncoupling agents, like blebbistatin, which have their pros and cons compared with 2,3-butanedione monoxime, given the randomized nature of this study, the agent chosen is not expected to alter the results. A bolus of 100 μL of the voltage dye di-4-ANEPPS (Biotium) was administered and when its distribution was not homogeneous, repeated administrations were made.

To excite di-4-ANEPPS, hearts were illuminated with a filtered green LED light source (maximum power output 58 W; maximum wavelength 524 nm) (Luminus Devices, Billerica, United States) through a plano-convex lens (LA 1951; focal length = 25.4 mm) (Thorlabs, New Jersey, United States) and a green excitation filter (D540/25X; Chroma Technology, Bellows Falls, United States). Three light sources were used to achieve homogeneous illumination. Both the Langendorff system and the protocol for performing optical mapping have been used and validated by other working groups (Langendorff, 1895; Efimov et al., 2004; Herron et al., 2012; Boukens and Efimov, 2014; Pavey et al., 2018; Swift et al., 2019; O’Shea et al., 2020).

**FIGURE 3:**
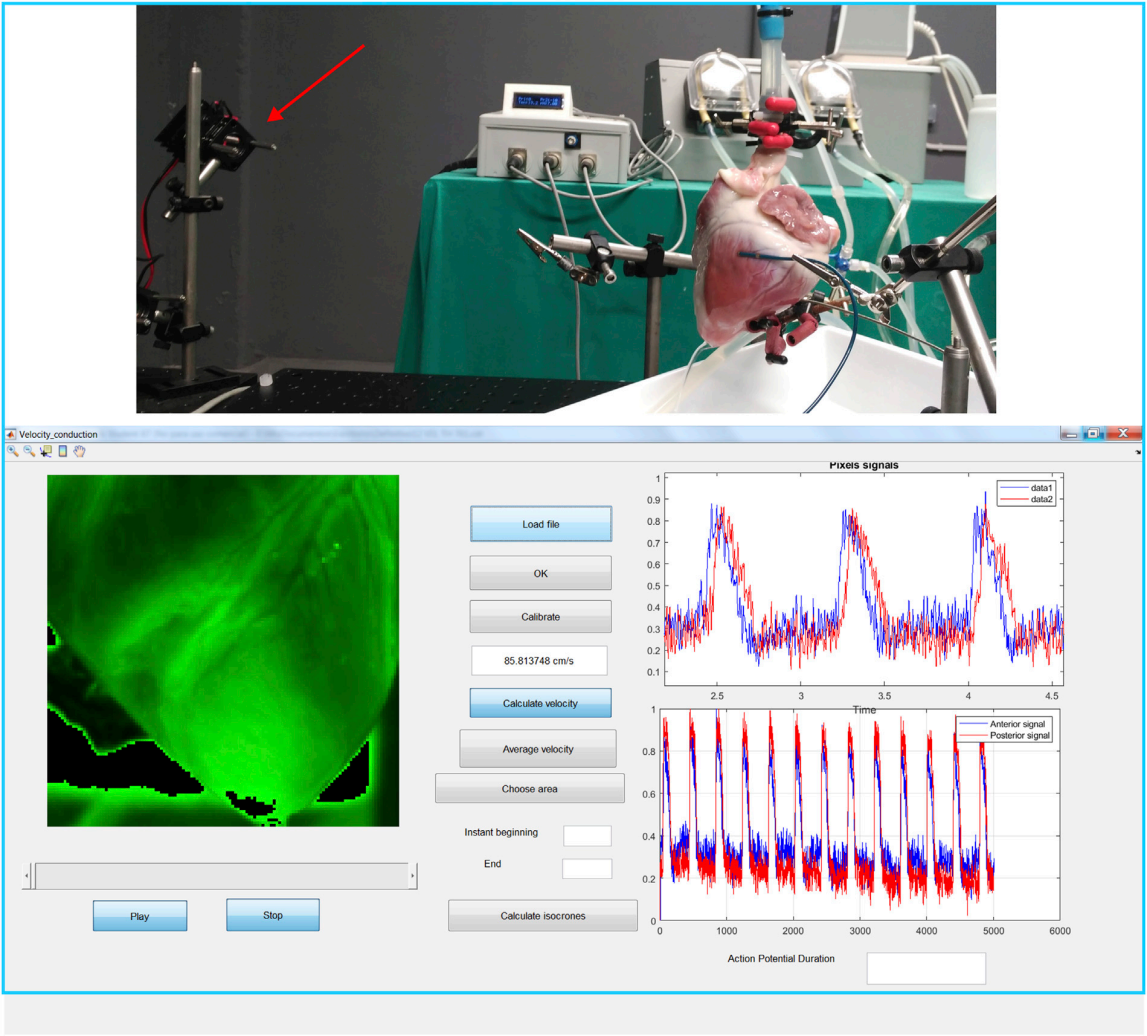
Epicardial optical mapping. Top: the layout of the equipment used for optical mapping (the red arrow points to the green light source). Bottom: a screenshot of the software used to perform image processing.

A custom software developed in the MATLAB (MathWorks, Natick, Massachusetts) environment was used to perform image recording (Electron Multiplygin Charge-Coupled Device Evolve 128 camera, 128 × 128 pixels, 24-μm square pixel, 16 bit) (Photometrics, AZ, United States) and processing during drive train pacing from the right ventricular epicardium at a 600 ms cycle length with rectangular pulses (amplitude 10 V and duration 2 ms) to ensure propagation was longitudinal to the epicardial fibers in the left ventricle (Figure 3). Conduction velocity (CV) was automatically estimated by calculating gradients of normal vector fields on isochronal propagation lines. Action potential duration (APD) of optical voltage signals was calculated at 80% repolarization. CV and APD were measured in the area covered by the camera where the HM and the HT were differentiated, the latter characterized by the presence of muscle fibers and fibrotic strands. Percentage changes in CV and APD were calculated by dividing the difference in the variable between the HT and HM by the value of the variable in the HM, i.e., (HT−HM)/HM , and compared between groups.

#### Histological study

Explanted hearts were fixed in cuvettes with formalin at room temperature. The scar was identified macroscopically and cut into transverse slices from apex to base for inclusion in paraffin. These slices were further cut into 4 μm-thick slices and were used for Masson’s trichrome staining and immunohistochemistry with the different primary antibodies against the different antigens (von-Willebrand factor; endothelial antigen CD31; and connexin 43) (Bio-Rad, CA, United States) after deparaffinization.

With Masson’s trichrome stain three types of tissue were differentiated: 1) DS, defined by the absence of myocytes; 2) HT, characterized by the presence of myocytes and strands of fibrosis that separate the cardiomyocyte bundles; and 3) HM, defined by normal cardiomyocytes with no intervening fibrosis. Analyses of the HT in the endocardial and epicardial halves of the myocardial thickness were performed independently. Measurements were made with a 20 mm × 40 mm transparent grid divided into 1250 squares to allow the conversion of semi-quantitative variables into quantitative variables.

The following semi-quantitative variables were determined for each HT compartment: fibrosis extension, the quantity of connexin 43, connexin 43 correct polarity, and cardiomyocyte viability (i.e. myocytes with no signs of hypertrophy, cytolysis, vacuolar degeneration or edema). For each of the semi-quantitative variables, a numerical value was obtained in such a way that +, ++, +++, ++++, and +++++ became 20, 40, 60, 80, and 100%. Since the scar was examined in different histological sections for each of the animals, a measurement of the extension of the HT on each of them was made. Depending on the area, each of the variables was subsequently weighted to obtain an average value of each variable per animal.

### Statistical analysis

Both the Mann-Whitney *U* test for independent samples and the Wilcoxon test for paired samples was used. The statistical analysis was performed with the Wilcox exact function, version 08–34, from the Package ‘exactRankTests’ in R version 4.0.5, which determines the exact *p*-value, even in the presence of ties. Statistical significance was established at a *p* < 0.05.

## Results

A total of 19 pigs were included and randomized to a treatment group (*n* = 10), to which CDCs were administered in the pericardial space by subxiphoid puncture, and to a control group (*n* = 9). The results obtained with the different techniques used (CMR, endocardial electrophysiological study, epicardial optical mapping, and histological study) are presented below.

All pigs survived the induction of the myocardial infarction and after balloon deflation they all had Thrombolysis In Myocardial Infarction Flow III. None of the animals died prematurely.

### Cardiac magnetic resonance

**FIGURE 4:**
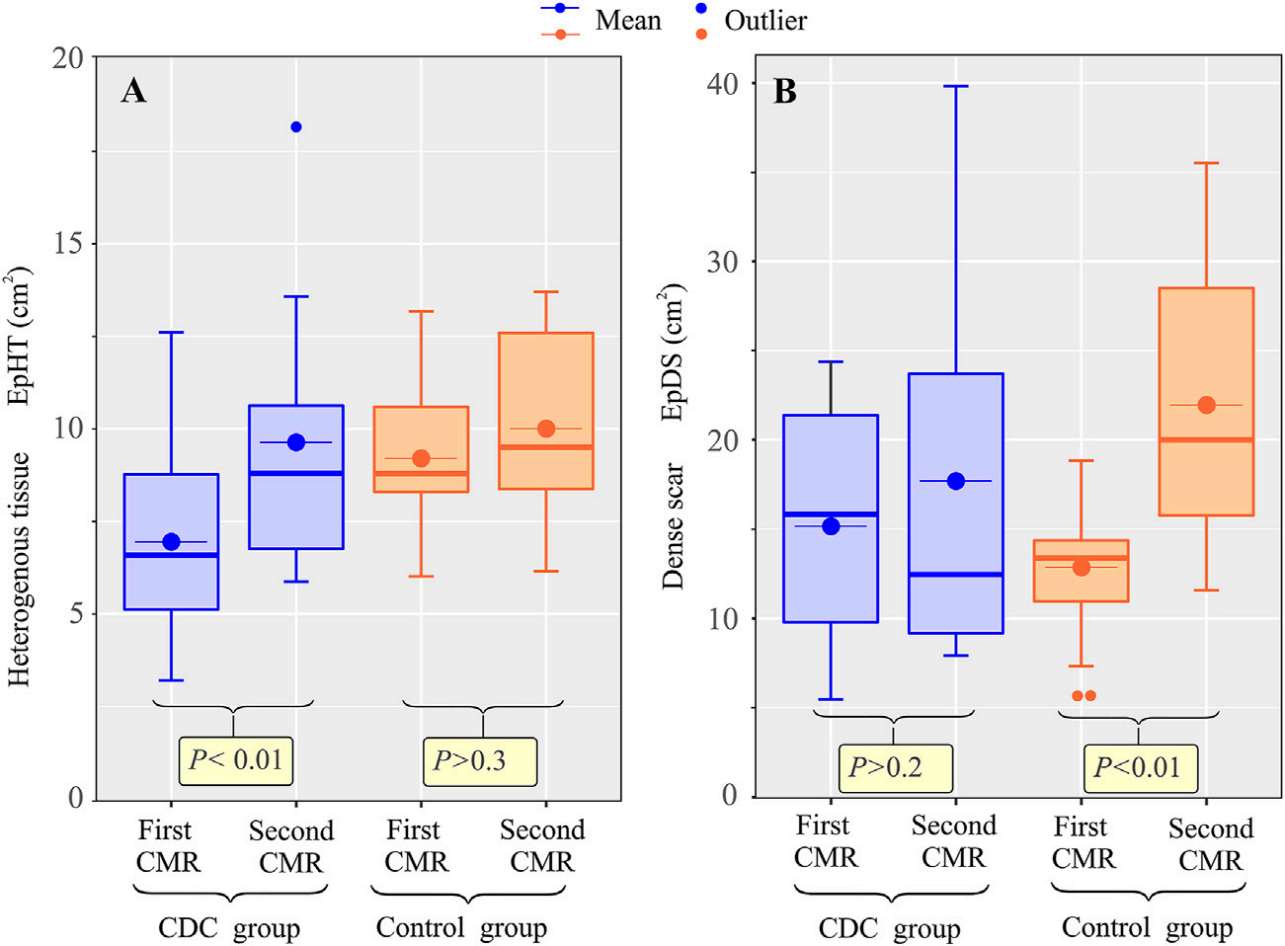
Effect of CDCs on epicardial ischemic scar composition. Boxplot showing the change in epicardial heterogeneous tissue, EpHT, **(A)** and dense scar, EpDS, **(B)** between the first and second CMR in the treatment and control groups.

**FIGURE 5:**
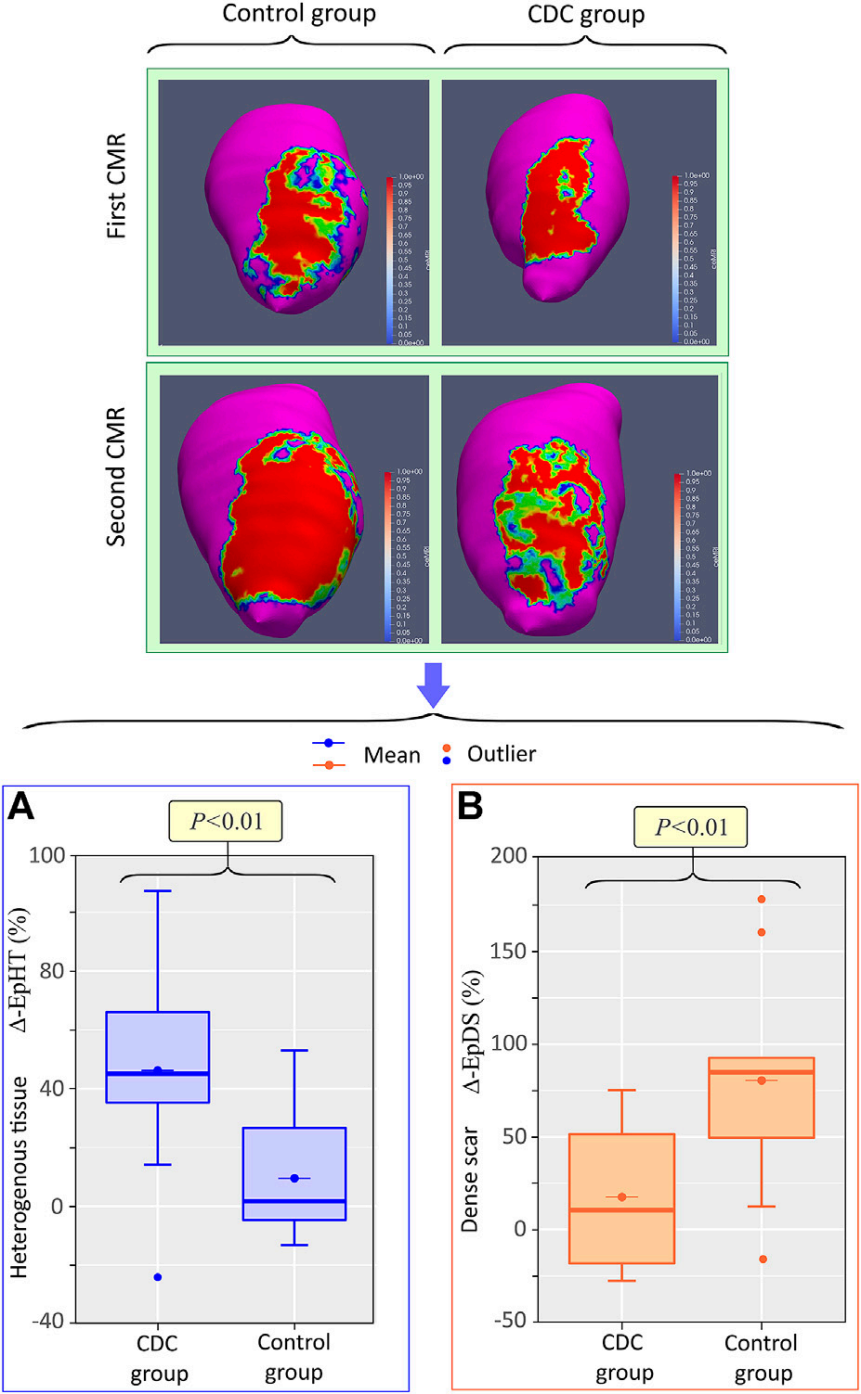
Intrapericardial CDCs block the conversion of heterogeneous tissue into dense scar in the epicardium. Top: epicardial signal intensity maps of a treatment and control animal in the first and second CMR. Red represents DS, magenta HM, and green-yellow HT. Bottom: boxplots showing the differences in relative percentage changes in epicardial heterogeneous tissue, EpHT **(A)** and dense scar, EpDS **(B)** Abbrv: CDC = cardiosphere-derived cells, CMR = cardiac magnetic resonance, ΔEpDS = percentage change in epicardial heterogeneous tissue, ΔEpHT = percentage change in epicardial heterogeneous tissue.

**FIGURE 6:**
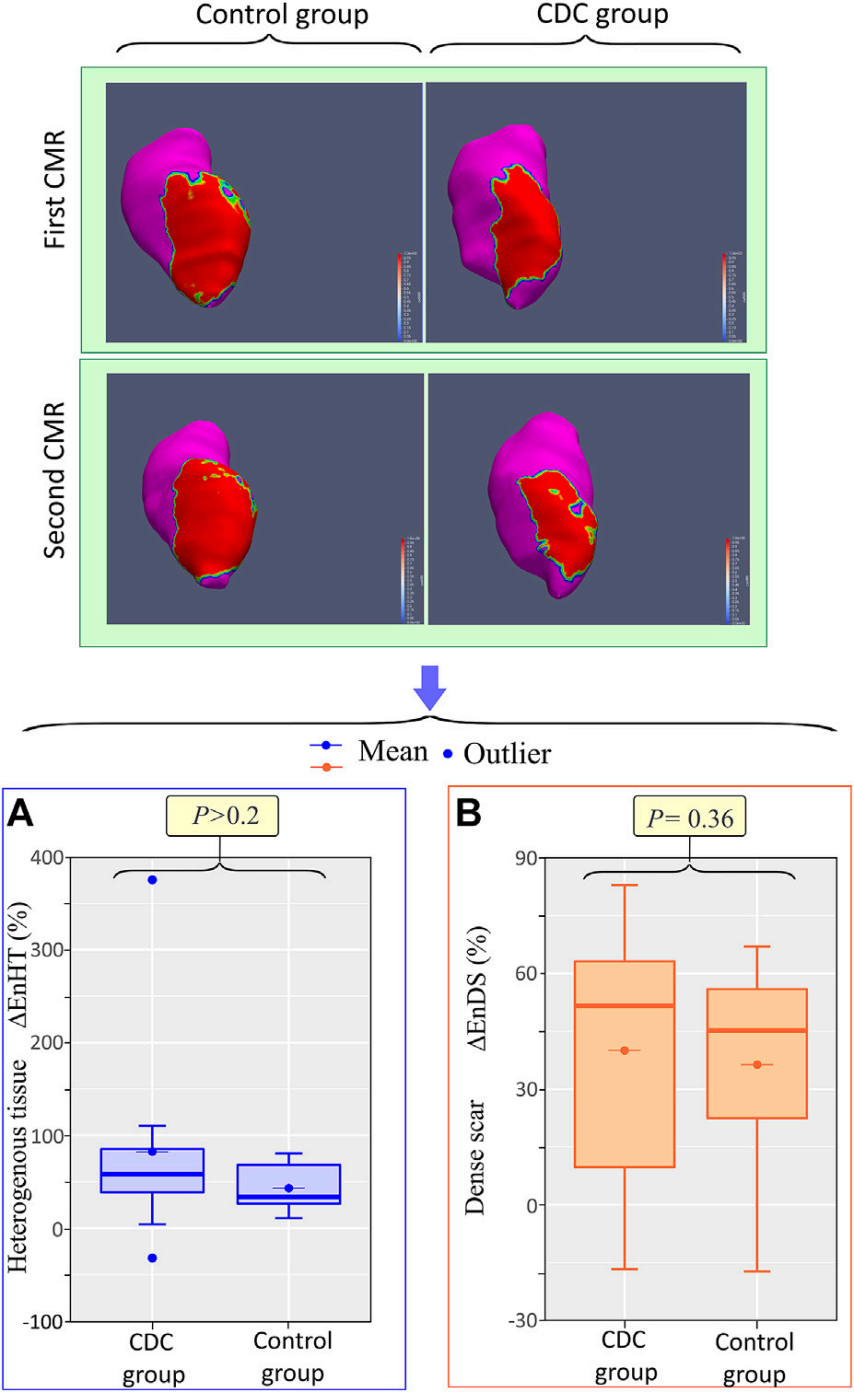
Intrapericardial CDCs do not modify endocardial scar composition. Top: endocardial signal intensity maps of a treatment and control animal in the first and second CMR. Red represents DS, magenta HM, and green-yellow HT. Bottom: Boxplots showing the differences in relative percentage changes in endocardial heterogeneous tissue, EnHT **(A)** and dense scar, EnDS **(B)** Abbrv: CDC = cardiosphere-derived cells, CMR = cardiac magnetic resonance, ΔEnDS = percentage change in endocardial dense scar, ΔEnHT = percentage change in endocardial heterogeneous tissue.

In the first CMR, there were no significant differences in ventricular volumes and dimensions, or scar extension and composition, between the treatment and control groups (Table 1). Between the first and second CMR, there was a significant increase in ventricular volume and TSM in both the treatment and control groups, with no significant change in LVEF in the control group (Table 2). At the endocardial level, there was a similar increase in HT and DS in both groups. However, at the epicardial level, total scar grew in both groups, but with significant differences in scar composition modification. In the treatment group, there was a significant increase in HT extension (median (Mdn) 6.6, interquartile range (IQR) [5.1, 8.8], vs. 8.8, [6.8, 10.6]; *p* < 0.01) (Figure 4A), while no significant change was observed in DS extension between the first and second CMR (Mdn 15.8, IQR [9.8, 21.4], vs. 12.5, [9.2, 23.7]; *p* > 0.2) (Figure 4B). In the control group, however, while no significant change was observed between the first and second CMR in HT extension [Mdn 8.8, IQR (8.3, 10.6), vs. 9.5, (8.4, 12.6); *p* > 0.3] (Figure 4A), a significant increase was observed in DS extension [Mdn 13.4, IQR (11.0, 14.4), vs. 20.0, (15.8, 28.5); *p* < 0.01] (Figure 4B). In addition, the epicardial relative percentage change in HT [Mdn 45.1, IQR (35.3, 66.0), vs. 1.7, IQR (−4.7, −26.7); *p* < 0.01] (Figure 5A) was significantly greater in the treatment group, whereas that of DS [Mdn 10.5, IQR (−18.3, 51.4), vs. 84.8, (49.5, 92.6); *p* < 0.01] (Figure 5B) was significantly greater in the control group. The endocardial relative changes in HT [Mdn 58.2, IQR (38.7, 85.2), vs. 33.6, (26.4, 68.2); *p* > 0.2] and DS [Mdn 51.6, IQR (9.8, 63.1), vs. 45.2, (22.4, 55.9); *p* = 0.36] did not differ between the treatment and control groups (Figure 6).

**TABLE 1:** Baseline characteristics of the treatment groups for the variables evaluated in the first CMR. Shown are the median values of the variables and the *p*-value of the contrast of hypotheses (Mann-Whitney test). LVEDV: end-diastolic left ventricular volume. EnDS: endocardial dense scar. EnHT: endocardial heterogeneous tissue. EpDS: epicardial dense scar. EpHT: epicardial heterogeneous tissue. LVESV: end-systolic left ventricular volume. LVEF: left ventricular ejection fraction. TSM: total scar mass. TMM: total myocardial mass.

Variable	Control group (*n* = 9)	CDC group (*n* = 10)	*p*
LVEDV (ml)	121 (120–138)	131 (122–145)	0.414
LVESV (ml)	95 (81–97)	93 (72–101)	0.968
LVEF (%)	30 (23–33)	29 (28–31)	0.549
TSM (g)	9.7 (8.9–10.6)	8.5 (6.8–9.0)	0.095
TMM (g)	64.4 (56.2–66.6)	58.3 (50.5–60.9)	0.438
EnDS (cm^2^)	18.9 (16.6–19.9)	14.9 (11.3–20.3)	0.278
EpDS (cm^2^)	13.4 (11.0–14.4)	15.8 (9.8–21.4)	0.549
EnHT (cm^2^)	3.7 (2.7–4.5)	3.1 (2.5–3.8)	0.500
EpHT (cm^2^)	8.8 (8.3–10.6)	6.6 (5.1–8.8)	0.113

**TABLE 2:** Evolution of left ventricular function, left ventricular volume, scar mass, and healthy myocardial mass as evaluated with CMR. Shown are the median values of the variables and the *p*-value of the contrast of hypotheses (Wilcoxon test). LVEDV = end-diastolic left ventricular volume. LVESV = end-systolic left ventricular volume. LVEF: left ventricular ejection fraction. TMM: total myocardial mass. TSM: total scar mass.

Variables	Baseline	Follow-up	*p*
Control group (*n* = 9)			
LVEDV (ml)	121 (120–138)	195 (171–209)	0.002
LVESV (ml)	95 (81–97)	124 (120–137)	0.002
LVEF (%)	30 (23–33)	31 (30–34)	0.125
TSM (g)	9.7 (8.9–10.6)	11.8 (9.5–12.6)	0.014
TMM (g)	64.4 (56.2–66.6)	85.4 (82.5–90.1)	0.002
CDC group (*n* = 10)			
EDV (ml)	131 (122–145)	196 (180–201)	0.001
ESV (ml)	93 (72–101)	138 (116–146)	0.001
LVEF (%)	29 (28–31)	26 (23–32)	0.042
TSM (g)	8.5 (6.8–9.0)	10.0 (8.4–11.3)	0.001
TMM (g)	58.3 (50.5–60.9)	88.7 (72.7–95.5)	0.001

### Endocardial electroanatomic mapping of the left ventricle

In the endocardial electroanatomical map, no statistically significant differences were observed in the DS and HT areas between groups (Table 3).

**TABLE 3:** Size of the endocardial scar evaluated with a high-density endocardial electroanatomic map. Shown are the median values ​​of the variables and the *p*-value of the hypothesis contrast (Wilcoxon test).

Bipolar electrogram amplitude				
Variables		Control group (*n* = 9)	CDC group (*n* = 10)	*p*
Endocardial scar (cm^2^)	0.0–0.5 mV	4.6 (2.3–9.5)	3.0 (1.7–6.9)	0.369
	0.5–1.5 mV	23.7 (20.1–24.9)	19.7 (17.3–23.7)	0.391

### Epicardial optical mapping

**FIGURE 7:**
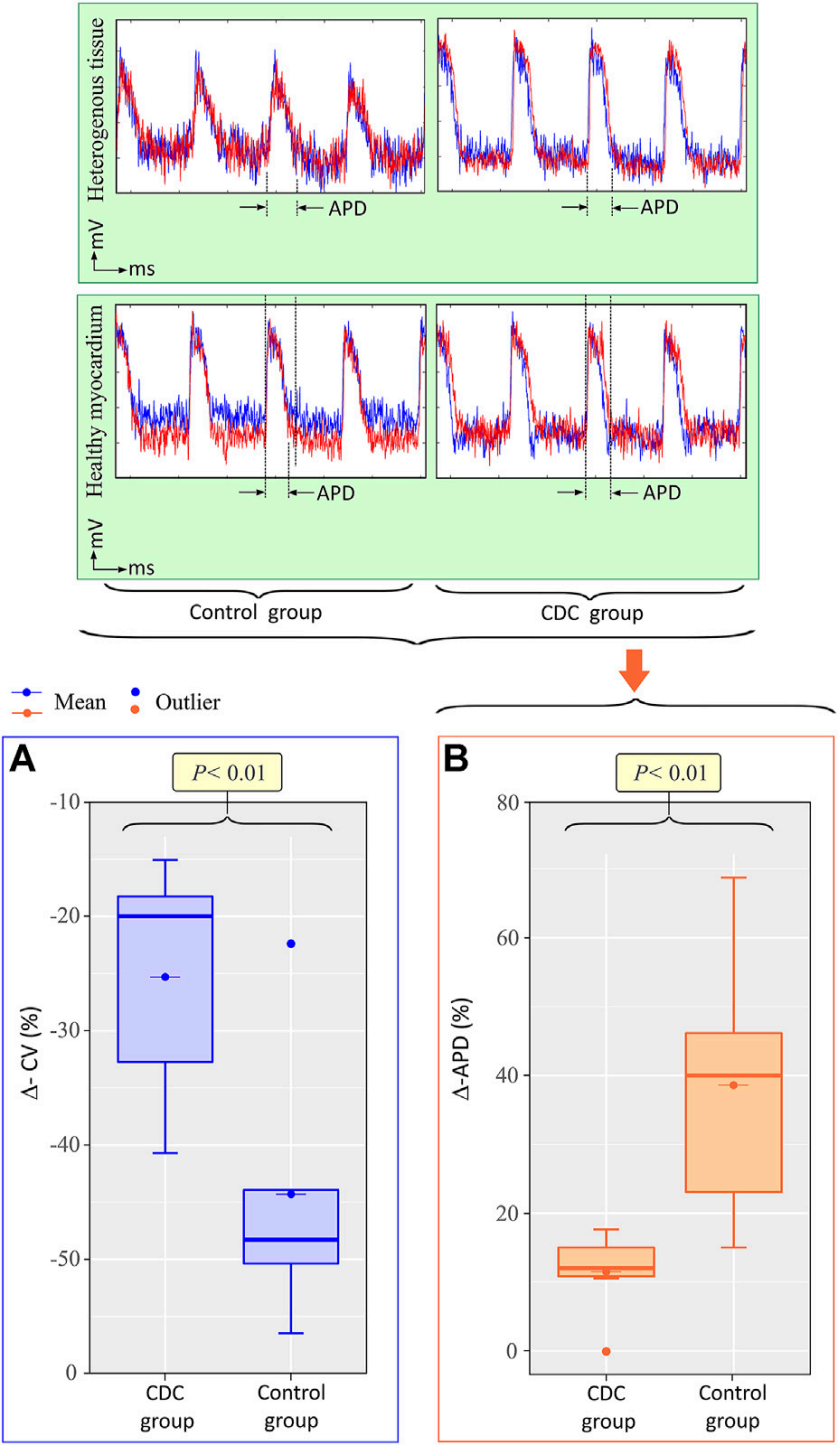
Intrapericardial CDCs produce epicardial electrical homogenization. Top: Epicardial optical mapping signal readings. Action potential duration (APD) is marked between arrows. Bottom: Boxplots showing the relative percentage changes in CV **(A)** and APD **(B)** between heterogeneous tissue and healthy myocardium. Abbrv: APD = action potential duration, CDC = cardiosphere-derived cells, ΔAPD = percentage change in action potential between heterogeneous tissue and healthy myocardium, ΔCV = percentage change in CV between heterogeneous tissue and healthy myocardium.

In the epicardial optical map, significant differences were observed between the treatment and control groups when the relative differences in CV [Mdn −20.0, IQR (−32.7, −18.2), vs. −48.3, (−50.4, −43.9); *p* < 0.01] (Figure 7A) and APD [Mdn 12.0, IQR (10.8, 15.0), vs. 40.0, (23.1, 46.2); *p* < 0.01] (Figure 7B) between HT and HM were compared. The treatment group presented less heterogeneity in electrophysiological properties at the epicardial level compared to the control group.

### Histology study

The epicardial HT of the treatment group had a lower percentage of fibrosis (IQR 5-30 vs. 20–55; *p* = 0.01), better cardiomyocyte viability (IQR 60–80 vs. 50–70; *p* = 0.02), and a higher quantity (IQR 72.5–90 vs. 65–80; *p* = 0.03) and polarity of connexin 43 (IQR 40–60 vs. 20–40; *p* = 0.02) than the control group. The epicardial HT was also better vascularized (IQR 60–65 vs. 50–60; *p* = 0.04) in the treatment group than in the control group. Boxplots of each of these variables can be consulted in the Supplementary Material. There were no significant differences between the endocardial HT of the treatment and control groups.

## Discussion

### Effect of CDCs on ventricular volumes and ejection fraction

Postinfarction ventricular volumes and total scar mass grew in both groups of pigs, with no significant differences between them. The acute loss of cardiomyocytes in infarction causes a sudden increase in myocardial loading conditions that induce ventricular remodeling (John Sutton and Sharpe, 2000). The results of this work are consistent with some preclinical and clinical studies where cellular therapy with CDCs did not show improvement in ejection fraction or ventricular volumes. Although the treatment group showed a statistically significant decrease in LVEF, the change is clinically not significant with a *p*-value close to the significance level (Malliaras et al., 2011; Makkar et al., 2012; Malliaras et al., 2014; Crisostomo et al., 2015; Kasai-Brunswick et al., 2017; Makkar et al., 2020). However, the lack of effect of the CDCs on ventricular volumes and function does not imply a lack of effect of cellular therapy on the arrhythmic substrate.

### Effect of CDCs on the scar remodeling

**FIGURE 8:**
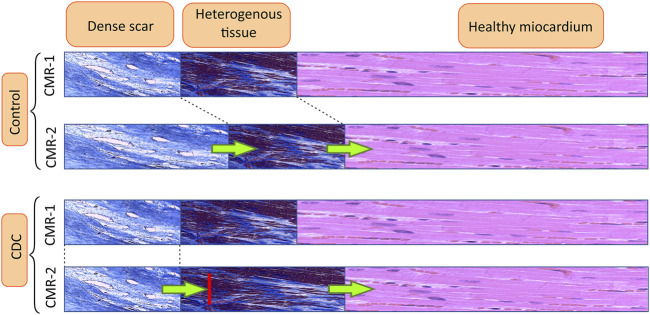
CDCs block the conversion of HT to DS in the epicardial scar. This diagram shows the evolution of the scar depending on the treatment group. Abbrv: CMR = cardiac magnetic resonance.

Intrapericardial administration of the CDCs did not affect the endocardial scar. The results of the endocardial electroanatomical mapping and the CMR were concordant in this respect. In contrast to the effect at the endocardial level, at the epicardial level, the modification of the scar was radically opposite for the 2 groups. The analysis of CMR data suggests that intrapericardial CDCs produce an anatomical impact, blocking the “compaction” of HT into DS at the epicardium by promoting myocyte viability and reducing fibrosis. In the treatment group, there is an increase in TSM between the two CMR studies but no change in DS. The increase in TSM is therefore due to the transformation of healthy myocardium into HT. In the control group, both TSM and DS increase but there is no change in HT. The increase in TSM is therefore partly due to the transformation of healthy tissue into HT and partly to the compaction of HT into DS (Figure 8).

The effect of CDCs limited to the epicardium that is observed can be speculated to be due to: 1) the close contact of the CDCs with the epicardium and/or, 2) the up-regulation of regeneration pathways that are mainly found in the epicardium and whose effects are restricted to the midmyocardial and epicardial level (Chimenti et al., 2010; Ibrahim et al., 2014; Sano et al., 2022).

The answer to how the CDCs block the transformation of the HT into a DS can be inferred from the histological study where it is evident that the HT of the treated pigs shows: 1) a lower percentage of fibrosis, 2) is better vascularized, and 3) contains better-preserved cardiomyocytes. These results are consistent with previous preclinical studies where allogeneic CDCs would localize to the scar border zone to “amplify” endogenous repair pathways. Thus, CDCs would upregulate cycling cardiomyocytes, favor angiogenesis, and suppress post-ischemic apoptosis to improve cardiomyocyte viability (Bersell et al., 2009; Chimenti et al., 2010; Baker et al., 2012; Malliaras et al., 2012; Tseliou et al., 2014). It can be speculated that the improved cardiomyocyte viability and vascularization are causally related to the antifibrotic effect. It has already been suggested that the increased density of vessels in the scar border zone partially cancels the expansion of the DS (Cohn et al., 2000; Tseliou et al., 2014). CDCs could also have a direct antifibrotic effect as has been shown with the expression of large amounts of metalloproteases of the matrix, although this has not been specifically tested in this work.

### Effect of CDCs on the endo- and epicardial electrophysiologic properties

CDCs produce a functional impact by reducing electrical remodeling of the scar border zone. By reducing the dispersion of the APD and CV between the HM and the HT, CDCs reduce electrical heterogeneity. These actions could modify and inactivate the appearance of the arrhythmic substrate in the epicardium. The higher quantity and better polarity of connexin 43 and the lower percentage of fibrosis in the epicardial HT of the treatment group could explain the greater epicardial electrical homogeneity. In this respect, a work studying the effect of connexin 43 expression on the electrophysiology of *ex-vivo* perfused mouse hearts, concluded that the electrical heterogeneity was determined by a heterogeneous electrical decoupling (Wiegerinck et al., 2008).

### Antiarrhythmic effect of CDCs

The establishment of a stable reentrant ventricular arrhythmia should have the following characteristics:

1) Inexcitable DS islands with a “critical mass” that allow the wavelength to adapt to the length of the circuit.
2) Heterogeneous tissue that facilitates the appearance of slow conduction and unidirectional block (electrophysiological heterogeneity).
3) Trigger (for example, a premature ventricular contraction).

The area of inexcitable tissue constitutes an obstacle that forces the depolarization front to divide and propagate independently on both sides of it. If the propagation front is unidirectionally blocked in one of the ways and advances only along the other, it will be able to surround the inexcitable area and reach the point where it was initially blocked, but in the opposite direction. If the depolarization front takes long enough (slow conduction) to allow this initially depolarized tissue to recover its excitability, it will again depolarize enabling conduction back into the circuit (reentry).

This work shows that CDCs block the transformation of HT into DS tissue. Several previous works have shown the relationship between the extension of the DS with ventricular arrhythmias. A previous work from our group that studied the arrhythmic substrate in a post-infarction model in pigs suggested the need for a “critical” size of inexcitable tissue for the establishment of stable ventricular tachycardia (Arenal et al., 2014).

In addition to the presence of unexcitable tissue and two conduction pathways, the initiation of reentry requires the existence of unidirectional conduction block. In cases where arrhythmic circuits are small the presence of slow conduction would be necessary to maintain reentry so that the cycle length is less than the circuit length. In this work, CDCs cause a greater homogeneity of the APD and the VC. The dispersion of repolarization increases the probability that, in the event of an early extrasystole, a unidirectional conduction block will be established and that the extrasystole will be conducted through the slow conduction pathway.

## Limitations

The first limitation is the low number of animals included. Secondly, cellular doses were defined by previous studies with intracoronary cell delivery, in which a balance was sought between achieving a biological effect and the risk of coronary obstruction. Given this risk is not a concern with intrapericardial cell delivery, we did not evaluate the persistence of CDCs in the pericardial sac, which constitutes another limitation. Concerns may also arise with the use of CDCs that block the conversion of HT into DS, thus producing heterogeneous scars with multiple islets of DS surrounded by HT. However, by halting the formation of a “critical mass” of unexcitable DS required for the establishment of an anatomic reentry a possible antiarrhythmic effect can be speculated. This conjecture, however, is at present difficult to test definitively. Finally, we did not test the inducibility of ventricular arrhythmia. However, it is open to speculation whether there would have been differences in inducibility given the effect of CDCs was limited to the epicardium, sparing the endocardial arrhythmic substrate, which could continue to sustain functional VT circuits. Unaltered endocardial substrate hampers the evaluation of the antiarrhythmic effect of CDCs on epicarcial VTs. There are two possible solutions to testing inducibility without being biased by the persistent endocardial substrate. One strategy would be to perform inducibility with simultaneous endo- and epicardial mapping to identify which of the VTs are epicardial in origin. The other strategy would be to use a swine model where the endocardium was spared. Limitations to the former strategy include that it is costly, time-consuming and that most VTs are not tolerated as to enable mapping (Connolly et al., 2000b; Antiarrhythmics versus Implantable Defibrillators (AVID) Investigators, 2007; Dinov et al., 2014), whereas the limitation to the latter strategy is the lack of a robust swine model with exclusive epicardial scaring. Given that the healing process of the myocardial infarction is ultimately a fibrotic reaction shared by other cardiomyopathies, we speculate that the changes produced by CDCs in the epicardium may be extensible to other heart diseases which predominantly affect the epicardium (i.e. arrythmogenic cardiomyopathy, postviral myocarditis, etc.) (Siontis et al., 2016).

Despite the above, these results are a proof-of-concept that CDCs administered into the pericardial sac modify epicardial scar remodelling (both anatomically and functionally). Although arrhythmia inducibility was not rested, our study showed that CDCs prevented the appearance of the electrophysiological changes that lead to a functional VT substrate (fibrosis, conduction velocity and action potentiall dispersion, dense scar growth). Further studies will be needed to unravel whether cellular therapy has a niche in the treatment of ventricular arrythmias with an epicardial substrate.

## Conclusion

Intrapericardial CDCs produce anatomical and functional changes of the epicardial arrhythmic substrate, which could have an anti-arrhythmic effect. The arrest of the fibrotic process with improved vascularisation and viability of epicardial HT cardiomyocytes, together with the increased amount and improved polarisation of connexin 43, could explain the anatomo-functional changes.

## Data Availability

The raw data supporting the conclusions of this article will be made available by the authors, without undue reservation.

## Abbreviations

APD, action potential duration; CDCs, cardiosphere-derived cells; CMR, cardiac magnetic resonance; CV, conduction velocity; DS, dense scar; EnDS, endocardial dense scar; EnHT, endocardial heterogeneous tissue; EpHT, epicardial heterogeneous tissue; EpDS, epicardial dense scar; HM, healthy myocardium; HT, heterogeneous tissue; ICD, implantable cardiac defibrillator; IQR, interquartile range; LVEDV, left ventricular end-diastolic volume; LVESV, left ventricular end-systolic volume; LVEF, left ventricular ejection fraction; Mdn, median; TMM, total myocardial mass; TSM, total scar mass; VT. ventricular tachycardia.
