# Supplementary material for: Intrapericardial cardiosphere-derived cells hinder epicardial dense scar expansion and promote electrical homogeneity in a porcine post-infarction model
Source: Front Physiol. 2022 Nov 15;13:1041348. doi: 10.3389/fphys.2022.1041348 (PMC9705343; doi:10.3389/fphys.2022.1041348)
Supplement: Supplementary file 1 [file DataSheet1.pdf]

Supplementary Material

Intrapericardial cardiosphere-derived cells hinder epicardial dense scar expansion and promote electrical homogeneity in a porcine post-infarction model

Alejandro Carta-Bergaz[1, 2], Gonzalo R. Ríos-Muñoz[1, 2, 3], Verónica Crisóstomo[2, 4], Francisco M. Sánchez-Margallo[2, 4], María J. Ledesma-Carbayo[5, 6], Javier Bermejo-Thomas[1, 2, 7], Francisco Fernández-Avilés[1, 2, 7], Ángel Arenal-Maíz[1, 2,*]

[1]Gregorio Marañón Health Research Institue (IiSGM), Department of Cardiology, Hospital General Universitario Gregorio Marañón, Madrid, Spain.
[2]Centre for Biomedical Research in Cardiovascular Disease Network (CIBERCV), Madrid, Spain.
[3]Departament of Bioengineering and Space Engineering, Universidad Carlos III de Madrid, Madrid, Spain.
[4]Jesús Usón Minimally Invasive Surgery Centre, Cáceres, Spain.
[5]Biomedical Image Technologies, ETSI Telecomunicación, Universidad Politécnica de Madrid, Madrid, Spain.
[6] CIBER-BBN, Instituto Salud Carlos III, Madrid, Spain.
[7]Medicine School, Universidad Complutense de Madrid, Madrid, Spain.

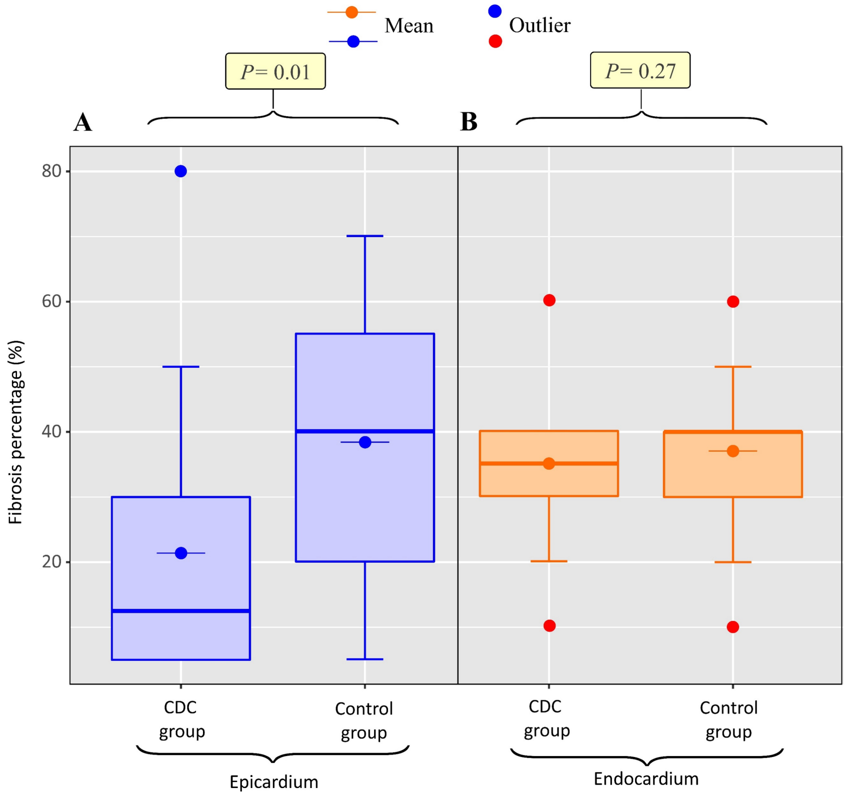

Figure S1. Boxplots of the percentage of fibrosis in the heterogeneous tissue in the inner and outer half of the ventricular wall of the treatment and control groups.

[*] Corresponding author
Email address: a.cartabergaz@gmail.com, arenal@secardiologia.es

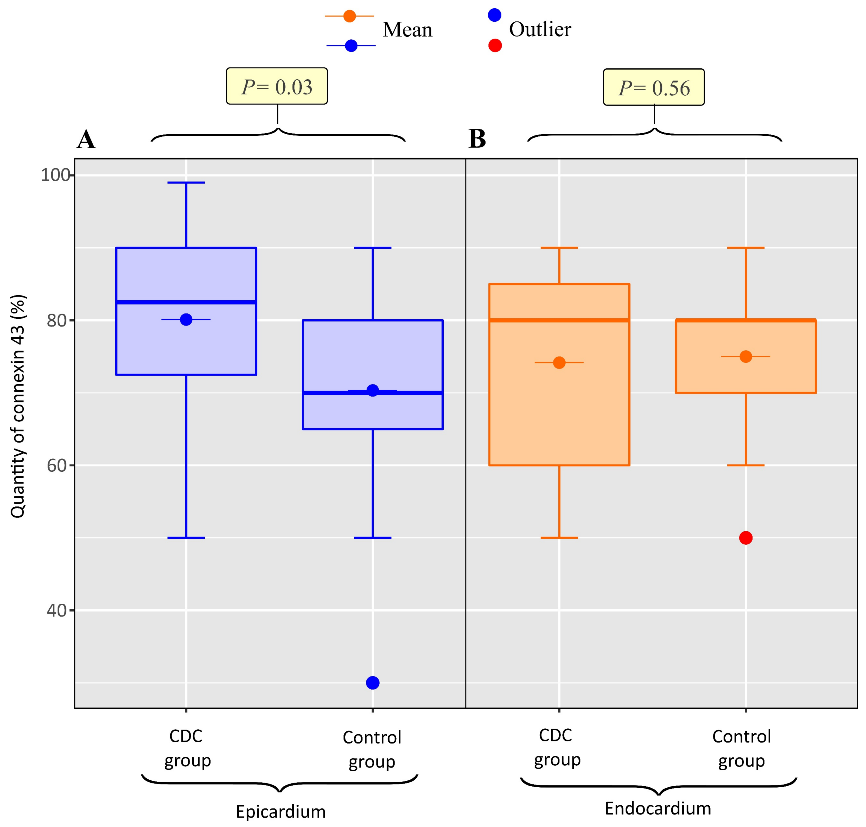

Figure S2. Boxplots of the quantity of connexin 43 in the heterogeneous tissue in the inner and outer half of the ventricular wall of the treatment and control groups.

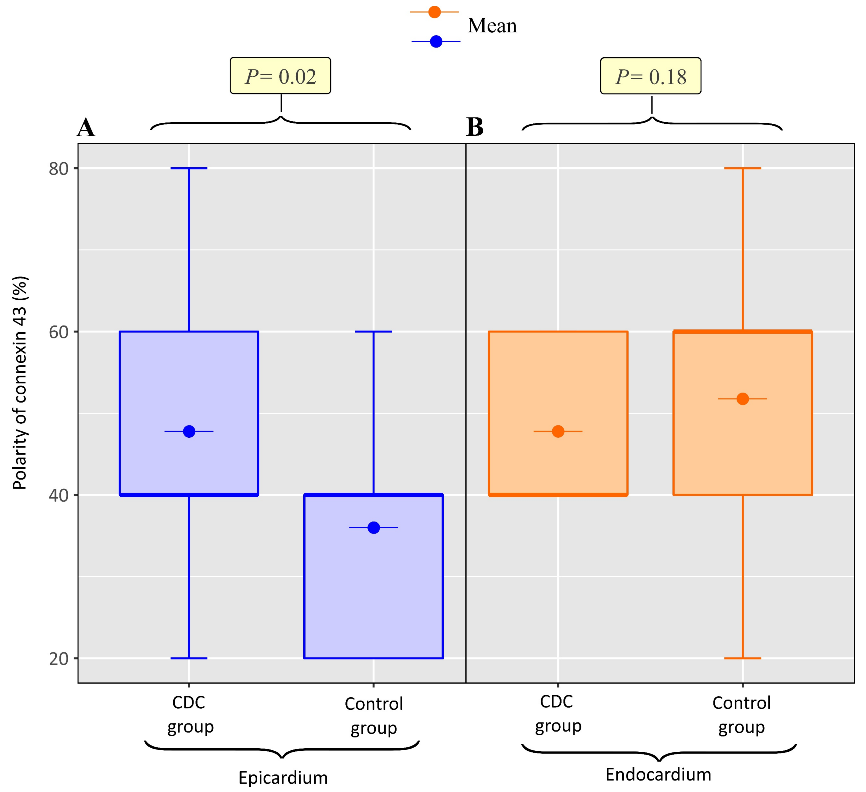

Figure S3. Boxplots of the polarity of connexin 43 in the heterogeneous tissue in the inner and outer half of the ventricular wall of the treatment and control groups.

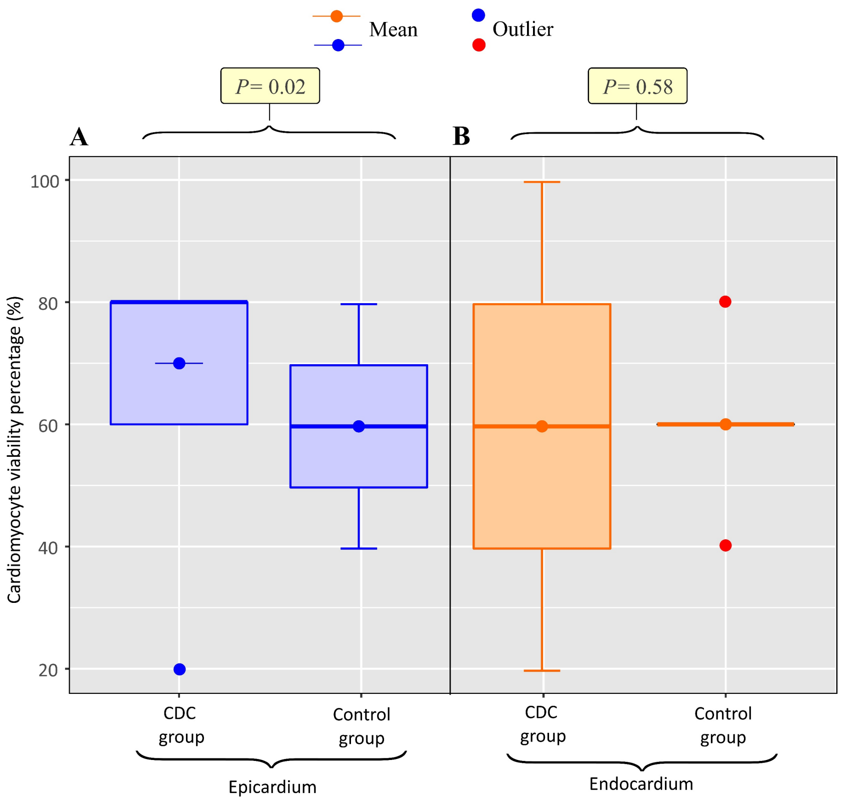

Figure S4. Boxplots of cardiomyocyte viability in the heterogeneous tissue in the inner and outer half of the ventricular wall of the treatment and control groups.

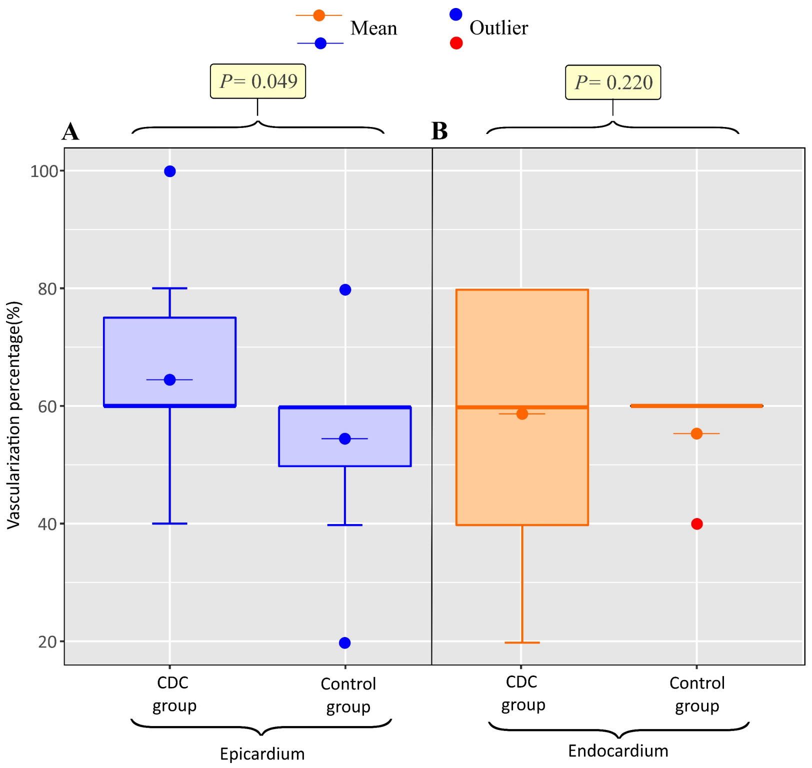

Figure S5. Boxplots of vascularization of heterogeneous tissue in the inner and outer half of the ventricular wall of the treatment and control groups.
